# Classification of Mental Health among Korean Vietnam War Veterans: A Latent Profile Analysis

**DOI:** 10.1093/geroni/igaa057.2183

**Published:** 2020-12-16

**Authors:** Hyunyup Lee, Carolyn Aldwin, Sungrok Kang, Xyle Ku

**Affiliations:** 1 Korea Military Academy, Seoul, Republic of Korea; 2 Oregon State University, Corvallis, Oregon, United States; 3 Korea Military Academy, Seoul, Seoul-t’ukpyolsi, Republic of Korea

## Abstract

We investigated the dimensional structure of mental health among aging Korean Veterans using latent profile analysis (LPA) on posttraumatic stress disorder symptoms (PTSD), late onset stress symptomology (LOSS), and psychosocial well-being (PWB). The Korean Vietnam War Veterans Study consists of 367 men (Mage=72, SD=2.66). LPA identified five classes of mental health as best fitting the data. Most men were in the normal (38%) and moderate distress (31%) groups, while smaller proportions were in the low affect (13%) and severe distress (7%) groups. The resilient group (12%) had low PTSD, medium LOSS, and high PWB, and were highest on optimism, positive appraisals of military service, and social support. Negative and positive aspects of mental health outcomes were on separate dimensions rather than on a single bipolar dimension. Service providers should attempt to both reduce Veterans’ negative psychological symptoms and increase psychosocial well-being. Part of a symposium sponsored by the Aging Veterans: Effects of Military Service across the Life Course Interest Group.

